# The nature of the T=T double bond (T = B, Al, Ga, In) in dialumene and its derivatives: topological study of the electron localization function (ELF)

**DOI:** 10.1007/s00894-019-4075-7

**Published:** 2019-07-04

**Authors:** Michal Michalski, Agnieszka J. Gordon, Slawomir Berski

**Affiliations:** Faculty of Chemistry, University of Wroclaw, 14 F. Joliot-Curie, 54-210 Wroclaw, Poland

**Keywords:** Triels, Boron group, Dialumene, Electron density, Electron localization function, ELF

## Abstract

The local electronic structure of the Al=Al bond was studied in dialumene and derivatives of dialumene in which the Al atoms were substituted by B, Ga, or In atoms. DFT calculations were performed using the B3LYP, B3PW91, PBE0, M06-L, and M06-2X functionals. Topological analysis of the electron localization function described the covalent bonds mentioned above using the disynaptic basins *V*_*i*=1,2_(B,B), *V*_*i*=1,2_(Al,Al), *V*(Ga,Ga), and *V*_*i*=1,2_(In,In). The basin populations were smaller than 4 *e*, as expected for a double bond: B=B 2.97 *e*, Al=Al 3.44–3.5 *e*, Ga=Ga 3.58 *e*, and In=In 3.86 *e*. The Al=Al, Ga=Ga, and In=In bonds were found to be intermediate in character between single and double bonds. Topological analysis of the *ρ*(*r*) field for dialumene showed a non-nuclear attractor along the Al=Al bond, with a pseudoatom basin population of 0.937 *e*. NBO analysis suggested that a double bond occurred only in the molecules containing Al, Ga, or In atoms. The character of the Ga=Ga bond was observed to be strongly dependent on the effective core potential used in the calculations.

## Introduction

**Scheme 1:**
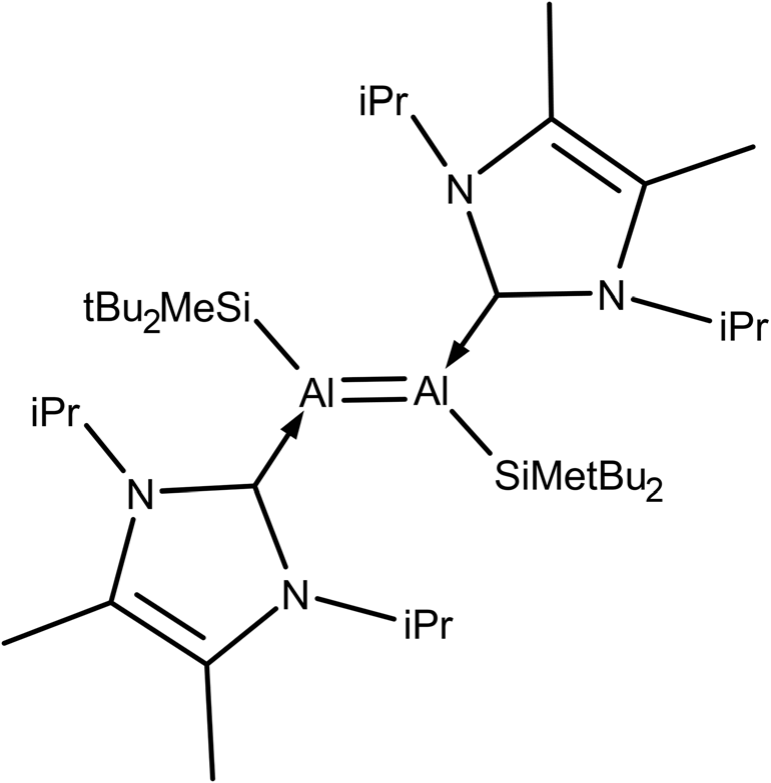
Approximate Lewis formula for the dialumene molecule (*Me –* methyl, *iPr –* isopropyl, *tBu –* tert-butyl)

Boron-containing compounds have fascinated researchers over the years [1–4], but research into homoatomic multiply bonded compounds has been fairly limited. There are several examples of boron molecules with homoatomic multiple bonding. Wang et al. [5] presented a neutral compound with the homoatomic B=B double bond, R(H)B=B(H)R (R = :C{N(2,6-iPr_2_C_6_H_3_)CH}_2_). A stable diborene at room temperature was reported by Braunschweig et al. [6]. The first stable molecule to be found to possess a formal double bond between aluminum atoms (i.e., Al=Al), dialumene, was synthesized and characterized by Bag et al. [7] using X-ray crystallographic and ^1^H NMR analysis (Scheme 1). This molecule possesses a trans-planar geometry in the solid state. Each aluminum atom adopts a trigonal planar coordination. Dialumene has been reacted with ethylene and phenyl acetylene in order to demonstrate the presence of an Al=Al double bond. The ^1^H NMR was used to show that a [2 + 2] cycloaddition reaction took place, leading to the formation of dialuminacyclobutane. Similarly, the reaction of phenyl acetylene with dialumene in toluene proceeds via both [2 + 2] cycloaddition and terminal C−H insertion. The most interesting observation made during those experimental studies was the unusual aluminum bond length. Single-crystal X-ray analysis showed that the homoatomic aluminum bond was 2.394(16) Å in length, which is outside the typical range for Al–Al single bonds [7].

Among the most recently developed computational methods for the electronic structure analysis of molecules are the electron localization function (ELF), *η*(*r*), as proposed by Becke and Edgecombe [8], and topological analysis of the ELF, as developed by Silvi and Savin [9]. A high value of the ELF corresponds to high electron localization. An ELF value of 0.5 corresponds to that of a uniform electron gas. Topological analysis of the ELF allows us to identify basins corresponding to attractors, which are chemically significant. The average population of an ELF basin is calculated by integrating the electron density over that basin [10].

The work reported in the current paper focused on the local nature of the homoatomic Al=Al bond in neutral dialumene. The electronic structures of similar homoatomic bonds (B=B, Ga=Ga, and In=In) were also investigated in order to establish general trends in the properties of interest. Electronic structures were studied by considering the topology of the ELF, the electron density, natural bond orbital (NBO) theory [11], and Wiberg bond indices (WBIs) [12].

## Computational details

The software Gaussian 09 (version E.01), G09 E.01 [13] was used to obtain the optimized structure of dialumene by applying the exchange-correlation functionals B3LYP [14], B3PW91 [15], PBE0 [16], M06-L, and M06-2X [17]. The standard Pople basis set [18, 19] 6-311++G(d,p) was used as implemented in G09. Molecules containing B, Ga, and In as their central atoms were studied at the DFT(M06-L) level using the 6-311++G(d,p) basis set for H, C, and N atoms. For gallium and indium derivatives, the LANL2DZ effective core potential (ECP) [20] was applied with the equivalent basis set. In order to examine the influence of the ECP type, other ECPs – ECP10MDF for the Ga atom, ECP28MDF for the In atom [21], and CRENBL for both metal atoms [22, 23] – were also used. Single-point energy calculations were subsequently carried out using the optimized structures. The LANL2DZ, ECP10MDF, ECP28MDF, and CRENBL ECPs were obtained from the EMSL Basis Set Library using the Basis Set Exchange software [24, 25]. Analysis of natural bond orbitals was carried out for all optimized structures using the Natural Bond Orbital (NBO 3.1) program in G09 [26]. All optimized structures were verified by vibrational analysis, which did not yield any imaginary frequencies. Additionally, wavefunction stability was tested for each optimized structure (keyword ‘stable’), and no instabilities were observed. All molecules were investigated in the singlet electronic state, using the closed-shell RHF formalism.

Topological analyses of *η*(*r*) and the electron density *ρ*(*r*) were carried out using the Multiwfn [27] and TopMod 09 [28] packages. When analyzing the Ga and In derivatives, extended wavefunction files were acquired using the ‘wfnx’ keyword. Topological analyses of *η*(*r*) and *ρ*(*r*) were implemented using a grid of points with a step size of 0.05 bohr. The total population of two *V*_*i*=1,2_(T,T) basins (T = B, Al, Ga, In) was estimated as the sum of the *V*_1_(T,T) and *V*_2_(T,T) basin populations.

Graphical representations of optimized structures, isosurfaces, and 2D ELF plots were prepared using the Chimera [29] and Multiwfn programs.

The Cambridge Structural Database (CSD; version 5.39, November 2017 [30]) was used to analyze B–B and Al–Al contacts.

## Results and discussion

### Geometric structures

**Fig. 1:**
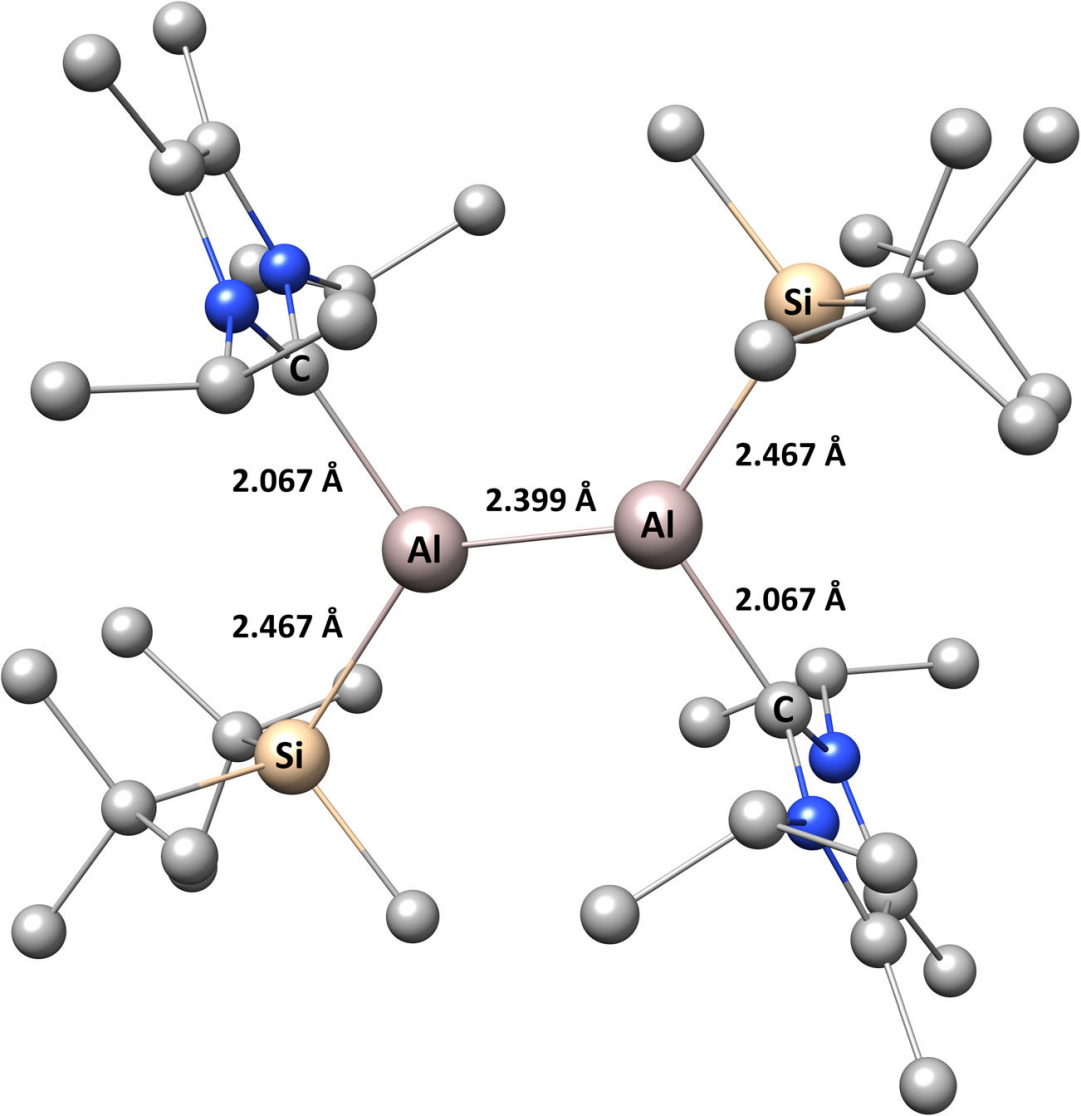
Optimized geometric structure of dialumene following optimization at the DFT(M06-L)/6-311++G(d,p) computational level (H atoms are omitted for clarity)

The structure of the dialumene molecule, which formally contains a double Al=Al bond, was optimized using density functional theory employing the 6-311++G(d,p) basis set and five exchange-correlation functionals: B3LYP, B3PW91, PBE0, M06-L, and M06-2X. Dialumene derivatives in which the Al atoms were substituted by B, Ga, and In atoms were optimized at the DFT(M06-L)/6-311++G(d,p) level. Selected bond lengths for all of the optimized compounds are presented in Table 1. The geometric structure of dialumene following optimization at the DFT(M06-L)/6-311++G(d,p) computational level is shown in Fig. 1.

**Table 1:** Optimized T=T, T–Si, and T–C bond lengths (in Å; T = B, Al, Ga, In) calculated using several DFT functionals and the 6-311++G(d,p) basis set

Bond	B	Al					Ga	In
	M06-L	B3LYP	B3PW91	PBE0	M06-L	M06-2X	M06-L	M06-L
T=T	1.628	2.435	2.441	2.431	2.399	2.389	2.392	2.757
T–Si	2.047	2.537	2.530	2.517	2.467	2.481	2.471	2.665
T–C	1.597	2.108	2.097	2.086	2.067	2.089	2.122	2.359

**Fig. 2:**
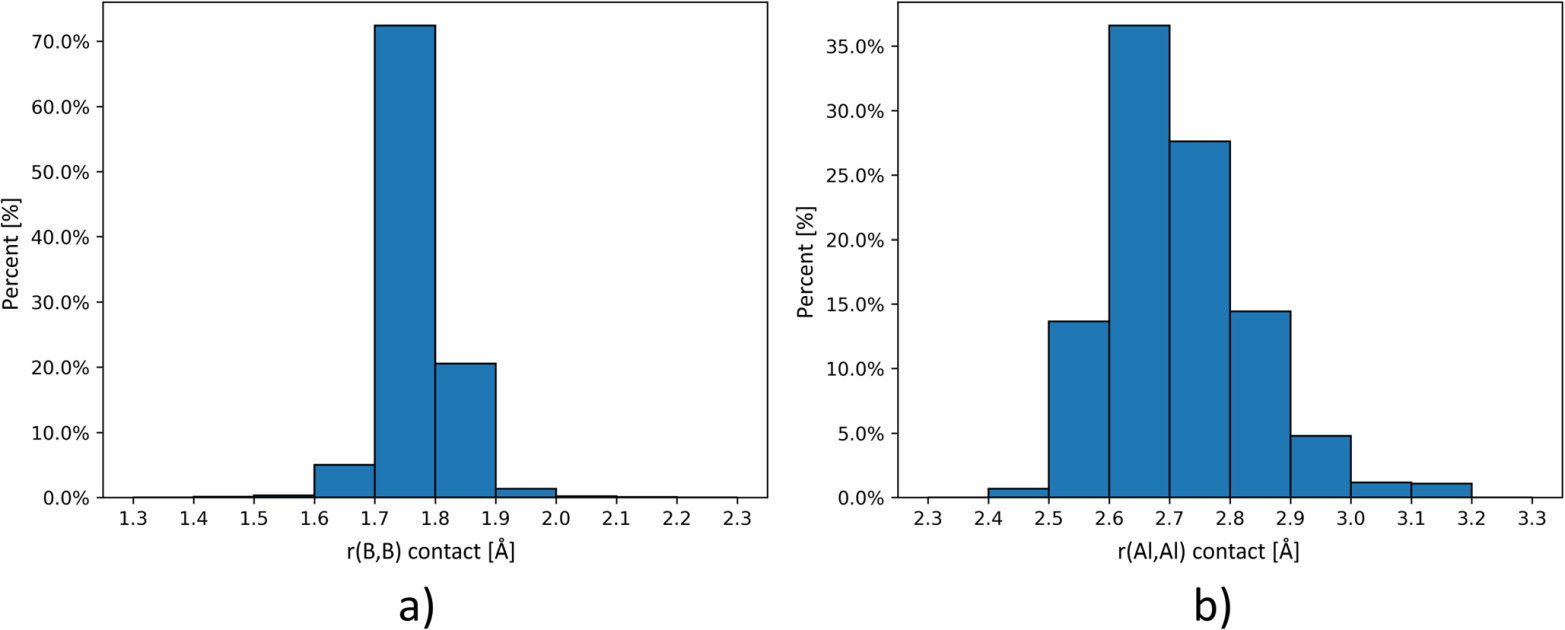
Histograms of the B–B (**a**) and Al–Al (**b**) contact distances for the molecules deposited in the Cambridge Structural Database

To facilitate comparison, we have also obtained B–B and Al–Al contact distances in crystal structures from CSD data, and the distributions of these contact distances are presented in Fig. 2. In the solid state, most of the B–B interactions occur at contact distances of between 1.7 and 1.8 Å (~70%), while the Al–Al interactions occur at contact distances of 2.6–2.8 Å (~65%). Optimized Al=Al bond lengths were between 2.389 Å (M06-2X) and 2.441 Å (B3PW91) and were noticeably shorter than the Al–Al contacts found in the CSD data (2.50–3.00 Å). The optimized B=B, Ga=Ga, and In=In bond lengths were 1.628, 2.392, and 2.757 Å, respectively. Thus, the B=B and Al=Al bonds in the molecules were found to be shorter than the contact distances of the most frequent triel interactions that occur in crystal structures. The reported B=B bond length is also much shorter than the single B–B bond in the planar B_2_H_4_ molecule (1.752 Å) [31]. The Ga=Ga bond length is 0.007 Å shorter than the Al=Al bond. This could be an effect of the LANL2DZ effective core potential used.

### Analysis of the bonding

Classical insights into the nature of the T=T bonding in the molecules of interest were obtained using the natural bond orbital method. The NBOs were studied using all the of density functionals mentioned above and the 6-311++G(d,p) basis set (see the “Computational details” section). The B, Ga, and In derivatives were studied at the DFT(M06-L)/6-311++G(d,p) level. The LANL2DZ effective core potential was applied to the Ga and In atoms.

Electron occupancy values for the NBOs in dialumene and its derivatives (T = B, Ga, In) are presented in Table 2. NBO pairs were obtained for the T=T (T = Al, Ga, In) bonds, so they exhibit double-bond character. The population of the Al=Al σ bond is 1.89 *e* or 1.90 *e*, and this bond is formed by the overlap of two *sp*^1.23–1.26^ natural hybrid orbitals. Occupancy of the π bond varies from 1.71 *e* to 1.77 *e*, and the bond is formed by pure Al *p* orbitals (at all computational levels). The double Ga=Ga bond is formed by the overlap of the *sp*^1.37^ hybrids in the σ bond with pure *p* orbitals (95%) in the π bond. In the In compound, the σ bond is formed by the overlap of the *sp*^1.53^ orbitals of the In atoms, and 87% of the π bond is contributed by the *p* orbital, a smaller proportion than for the corresponding Ga=Ga bond. The most interesting results were obtained for the B–B bond. NBO analysis showed that only σ bond was present, formed through the overlap of *sp*^1.30^ and *sp*^1.31^ hybrids (43% *s* orbital and 57% *p* orbital). There was no indication of π bond. Such a quantitative difference in bonding nature between the formal B=B bond and the NBO results for the T=T (T = Al, Ga, In) bonds should be reflected in topological analyses of the *η*(*r*) and *ρ*(*r*) fields.

**Table 2:** Calculated electron occupancy values for the σ and π bonds for the T=T bonds in the dialumene molecule (T = Al) and its derivatives (T = B, Ga, In), as well as Wiberg bond indices (WBI)

Parameter	B	Al					Ga	In
	M06-L	B3LYP	B3PW91	PBE0	M06-L	M06-2X	M06-L	M06 L
σ bond	1.86	1.90	1.90	1.90	1.89	1.90	1.89	1.82
π bond	–	1.75	1.75	1.75	1.71	1.77	1.70	1.71
WBI	1.55	1.66	1.65	1.65	1.60	1.66	1.57	1.45

Calculations were carried out using DFT employing the B3LYP, B3PW91, PBE0, M06-L, and M06-2X electron density functionals and the 6-311++G(d,p) basis set

Moving down the periodic table, the calculated Wiberg bond indices (WBIs) ranged from 1.55 (B=B) to 1.45 (In=In). Thus, there was no indication of a pure double bond; the bonds appeared to be intermediate in character between single and double bonds (see Table 2). The calculated WBI for the Al=Al bond ranged between 1.60 (M06-L) and 1.66 (B3LYP and M06-2X). A slightly smaller value (1.55) was obtained for the B=B bond, similar to that calculated for the Ga=Ga bond (1.57). Thus, the B=B bond was similar in character to the Al=Al bond, but with less double-bond character. It is worth emphasizing that the WBI analysis did not point to any qualitative difference between B=B and the other T=T (T = Al, Ga, In) bonds, in contrast to what was found using the NBO method.

**Fig. 3:**
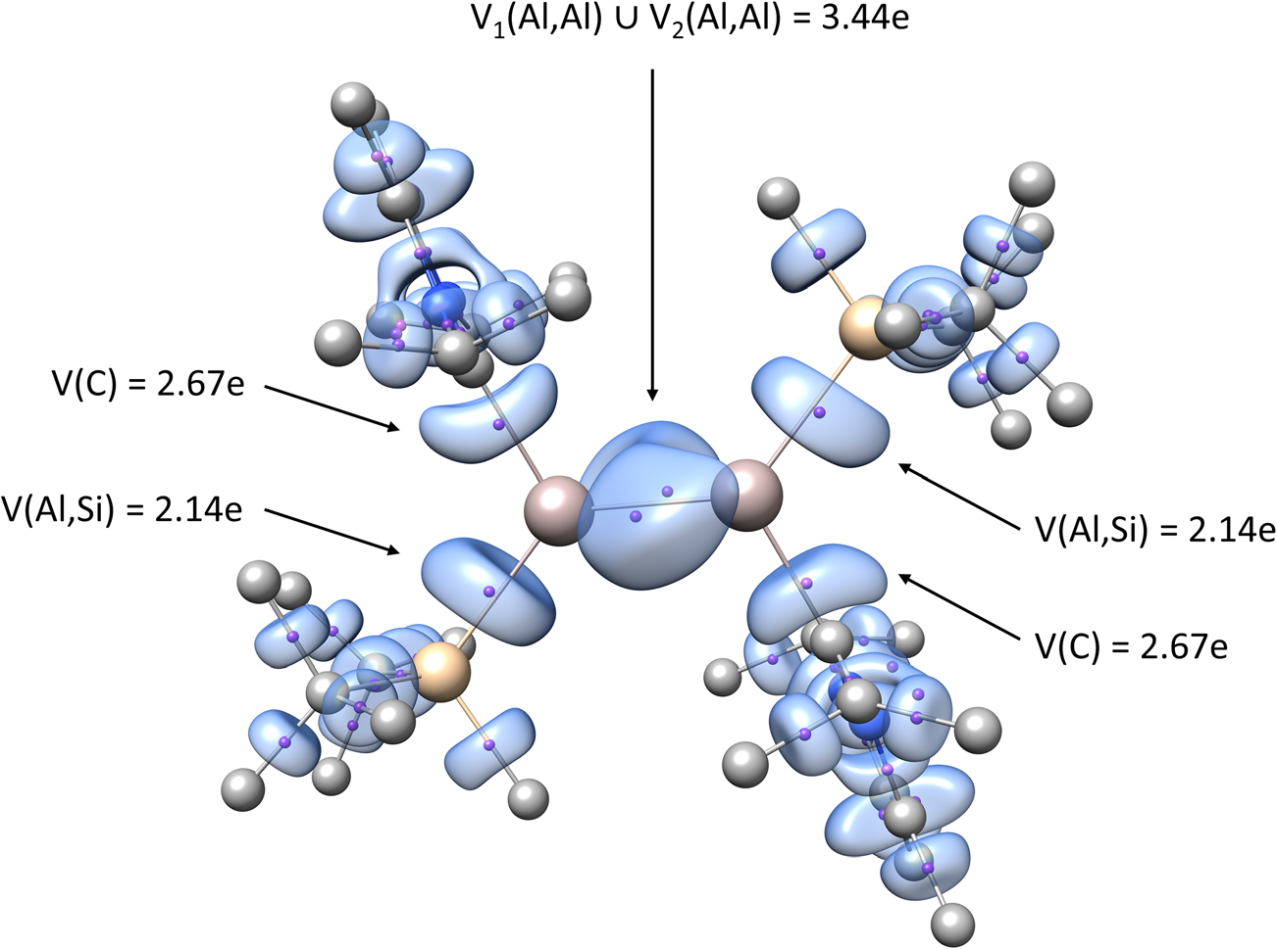
Graphical representation of the ELF localization domains (ELF = 0.795) for dialumene, labeled with the positions of valence attractors and basin populations. Calculations were carried out at the DFT(M06-L)/6-311++G(d,p) computational level. Valence domains for hydrogens are omitted from the graphic for the sake of clarity

The next step in our investigation of the local electronic structure of dialumene and its derivatives with B=B, Ga=Ga, and In=In bonds was to perform topological analysis of the ELF. Figure 3 illustrates the localization domains for the Al compound; the positions of the valence attractors are marked. The mean electron populations, $\overline{N}$, for the core and valence basins in all of the molecules are presented, along with the topological bond orders, in Table 3.

**Table 3:** Basin population values, $\overline{N}$ (*e*), standard deviations of $\overline{N}$ (*σ*), and topological bond orders (TBOs) for the T=T bonds in the dialumene molecule (T = Al) and its derivatives (T = B, Ga, In)

Parameter	B	Al					Ga	In
	M06-L	B3LYP	B3PW91	PBE0	M06-L	M06-2X	M06-L	M06-L
*V*_1_(T,T)	1.60	1.75	1.74	1.74	1.72	1.77	3.58	1.92
*V*_2_(T,T)	1.37	1.75	1.75	1.74	1.72	1.77		1.94
*V*(T,Si)	2.20	2.12	2.13	2.13	2.14	2.12	2.15	2.06
*V*(T,C)^a^	2.64	2.61	2.62	2.60	2.67	2.59	2.60	2.58
*σ*	0.97 0.93	0.98 0.98	0.99 0.99	0.98 0.99	0.98 0.99	0.85 0.85	1.18	1.00 1.00
TBO	1.49	1.75	1.75	1.74	1.72	1.77	1.79	1.73

The calculations for dialumene were performed using wavefunctions approximated with different DFT functionals and the 6-311++G(d,p) basis set. Calculations for B=B, Ga=Ga, and In=In were performed at the DFT(M06-L)/6-311++G(d,p) computational level

^a^For B=B V(B,C), otherwise V(C)

The electronic structure of dialumene was found to be characterized by 48 core and 138 valence attractors (local maxima of the *η*(*r*) field). Similar numbers of attractors were obtained for the dialumene derivatives with B, Ga, and In atoms. The Al=Al bond is characterized by two bonding disynaptic attractors with disynaptic basins *V*_1_(Al,Al) and *V*_2_(Al,Al), respectively. The concept of basin synapticity has been explained by Silvi [32]. Each *V*_*i*=1,2_(Al,Al) localization basin has a surface in common with two C(Al) core basins. According to the interpretation proposed by Silvi and Savin [33], shared-electron interactions such as covalent, dative, and metallic bonds occur when there is at least one bond attractor between the core attractors of the atoms involved in the bond. Thus, the localization of the *V*_*i*=1,2_(Al,Al) attractors yields proof that the Al–Al interaction is covalent, with shared electron density. Two bonding attractors *V*_*i*=1,2_(Al,Al) are localized approximately perpendicular to the neighboring bonds and in the regions with high electron localization. Pauli repulsion between electron clouds from the Al=Al, Al–C, and Al–Si bonds in those areas is bound to be smaller than that in the Al–C, Al=Al, and Al–Si plane. Two attractors are observed, which may correspond to two Al=Al bonds (σ, π). In a seminal paper on the electronic structures of delocalized bonds, Savin et al. [10] showed two *V*_*i*=1,2_(C,C) attractors for ethylene localized in the C=C bonding region. However, the localization of multiple bonding attractors is not regarded as proof of multiple bonding [34]. The topology of ELF in the region of the Al–Al interaction is indicative of a local symmetry rather than a double bond. For the B=B and In=In bonds, the local topology of ELF again includes two bonding disynaptic attractors, *V*_*i*=1,2_(T,T). The Ga=Ga bond is an exception, as it includes only a single bonding attractor, *V*(Ga,Ga). Thus, the B=B, Ga=Ga, and In=In bonds exhibit covalent character, as also observed for the Al=Al bond.

The total basin population for the Al=Al bond (see the “Computational details” section) varies from 3.44 *e* to 3.50 *e*, depending on the DFT functional used (see Table 3). Thus, the topological approach partially supports the idea that this is a double bond, bearing in mind that the formal value of 4 *e* for such bond is idealized anyway. The basin population for the C=C bond in ethylene, a prototypical molecule with a double bond, is found to be 3.40 *e* at the DFT(M06-L)/6-311++G(d,p) computational level, suggesting that we should expect some similarity between the Al=Al and C=C bonds. For the B=B, Ga=Ga, and In=In bonds, as shown in Table 3, the total population of the bond increases from 2.97 *e* (B) to 3.86 *e* (In). The value of 3.58 *e* is concentrated in the single-valence bonding basin *V*(Ga,Ga). It is worth emphasizing that, for the In–In interaction, the bond character interpreted from the ELF is very close to the formal character (i.e., a double bond). It is possible that more polarizable atoms (Ga: 8.12 Å^3^, In: 9.1 Å^3^) [35] containing larger numbers of diffuse atomic orbitals contribute more electron density to localization basins during the bond formation process. Basins with larger populations correspond to more covalent chemical bonds.

The topological bond order, TBO (see Table 3), for the B=B bond is 1.49. For Al=Al, the TBO value is not very sensitive to the functional used: it ranges between 1.72 and 1.77. The TBO for B=B is 1.49 (M06-L), which is 0.23 smaller than that calculated for Al=Al (M06-L), suggesting rather different bond character. The TBOs for Ga=Ga and In=In are similar to that for Al=Al (1.79 and 1.73, respectively). Thus, the TBOs confirm the double-bond character of the Al=Al, Ga=Ga, and In=In interactions. The B=B bond is different; based on the topological analysis of ELF, it should not be formally categorized as a double bond.

The standard deviation, *σ*, obtained for a localization basin can be interpreted as a measure of the electron delocalization [36]. The values of *σ* for the T=T bonding basins are presented in Table 3. The *V*_*i*=1,2_(B,B), *V*_*i*=1,2_(Al,Al), and *V*_*i*=1,2_(In,In) basins yield values of between 0.85 and 1. The *V*(Ga,Ga) basin yields a significantly larger *σ* value, 1.18. This might be due to the fact that the Ga=Ga bond is described only by a single basin, *V*(Ga,Ga). Since these *σ* values are smaller than those for the N, the electron density is well localized in the T=T bonding region. This feature is typical of standard covalent bonds, i.e., those with basin populations much larger than 1 *e*.

As described by Bag et al. [7], the double Al=Al bond in dialumene is bound to two stabilizing substituents at each end – the silyl group (tBu_2_MeSi) and *N*-heterocyclic carbene ((CH_3_)_2_C_3_N_2_(iPr)_2_). We therefore decided that it was worth investigating the nature of the C–Al and Si–Al bonds from an ELF topological perspective. The C–Al bond is formally (i.e., in the Lewis structure presented in Scheme 1) a covalent dative bond, where two electrons are donated to the Al valence shell (C → Al). Our study was extended to examine the local electronic structures of the T–C (T = B, Al, Ga, In) bonds in all of the derivatives of dialumene.

For the molecule in which the Al atoms are substituted by B atoms, topological analysis of the *η*(*r*) function of the C–B bond reveals a single disynaptic bonding attractor, *V*(B,C). Thus, the bond has covalent character, with electron density shared by the B and C atoms. Interestingly, different local ELF topologies are observed for the molecules with Al=Al, Ga=Ga, and In=In bonds. The C → Al bond is characterized by the monosynaptic nonbonding attractor *V*(C) only; no disynaptic attractor *V*(T,C) is present. Thus, based on Silvi and Savin’s interpretation [33], the C → Al bonds are not classically covalent bonds with a shared electron density. Those bonds are more of the donor–acceptor type, where electron density of the carbon atom is donated to the valence shell of the heavier triel atom (Al, Ga, or In). The results support Bag et al. interpretation of the dative character of the C–Al bond [7].

Analysis of atomic contributions to the localization basins proposed by Raub and Jansen [37] partially confirms this interpretation. Atomic contribution results are shown in Table 4. The contribution of the boron atom to the *V*(B,C) basin of the B–C bond is 0.21 *e* (~8%) smaller than that of the C atom, 2.42 *e* (~92%). The covalent B–C bond is polarized towards the C atom, and the polarity index *p*_CB_ is 0.84. The value of the polarity index ranges between 0 for homopolar bonds and 1 for idealized ionic bonds. The bond has high covalent and polarized character. Analysis of the molecules containing Al, Ga, and In atoms shows that the contribution of the metal to the *V*(C) basin, 0.11 *e*, is smaller than that found for the B-substituted derivative, 0.21, and the participation of the C atom is greater: 2.47–2.54 *e* (4%) for Al, 2.46 *e* (4%) for Ga, and 2.44 *e* (4%) for In. Such a result confirms that *V*(C) is a monosynaptic basin, since the atomic contribution, 4%, is very small – approximately half that calculated for the covalent B–C bond. Nevertheless, to the best of our knowledge, there is no study in which the atomic contribution is correlated with topological characteristics such as synapticity. In summary, analysis of atomic contributions to the *V*(C) and *V*(B,C) basins shows that the binding between the triel and C atoms is dominated by electron density from the carbon atom, despite the different characteristics obtained with the synapticity concept.

**Table 4:** Atomic contributions (in *e*) to the *V*(T,C) and *V*(C) localization basins in dialumene and its derivatives, T = (B, Al, Ga, In)

Parameter	B	Al					Ga	In
	M06-L	B3LYP	B3PW91	PBE0	M06-L	M06-2X	M06-L	M06-L
$\overline{N}$[T|V(C)]	–	0.11	0.11	0.11	0.11	0.11	0.11	0.11
$\overline{N}$[C|V(C)|	–	2.47	2.48	2.46	2.54	2.46	2.46	2.44
$\overline{N}$[T|V(T,C)]	0.21	–	–	–	–	–	–	–
$\overline{N}$[C|V(T,C)]	2.42	–	–	–	–	–	–	–

Calculations were performed using wavefunctions approximated by the DFT method employing various electron density functionals and the 6-311++G(d,p) basis set

To gain further insight into the nature of the T=T bonds, topological analysis of the *ρ*(*r*) field developed by Bader [38] was carried out. The values calculated for the (3,−1) bond critical points (BCPs) of the T=T bonds, i.e., *ρ*_(3,−1)_(*r*), and the Laplacian of the electron density, ∇^2^*ρ*_(3,−1)_(*r*), are collected in Table 5.

**Table 5:** Calculated values for the bond critical points (BCPs) of the T=T (T = B, Al, Ga, In) bonds in the dialumene molecule (T = Al) and its derivatives (T = B, Ga, In)

Parameter	B	Al					Ga	In
	M06-L	B3LYP	B3PW91	PBE0	M06-L	M06-2X	M06-L	M06-L
*ρ*_(3,−1)_(*r*) (*e*/au^3^)	0.152^b^	0.058^a^ 0.057^b^ 0.057^b^	0.056^a^ 0.055^b^ 0.055^b^	0.057^a^ 0.056^b^ 0.056^b^	0.058^a^ 0.057^b^ 0.057^b^	0.059^a^ 0.058^b^ 0.058^b^	0.063^b^	0.046^b^
∇^2^*ρ*_(3,-1)_(*r*) (*e*/au^5^)	−0.308^b^	−0.080^a^ −0.027^b^ −0.027^b^	−0.075^a^ −0.022^b^ −0.022^b^	−0.077^a^ −0.017^b^ −0.017^b^	−0.081^a^ −0.007^b^ −0.007^b^	−0.084^a^ −0.019^b^ −0.019^b^	0.003^b^	0.040^b^

Calculations were performed using DFT employing several electron density functionals and the 6-311++G(d,p) basis set

Here, *ρ*_(3,−1)_(*r*) is the electron density at the BCP and ∇^2^*ρ*_(3,−1)_(*r*) is the Laplacian of the electron density at the BCP

^a^NNA (non-nuclear attractor)

^b^BCP (bond critical point)

**Fig. 4:**
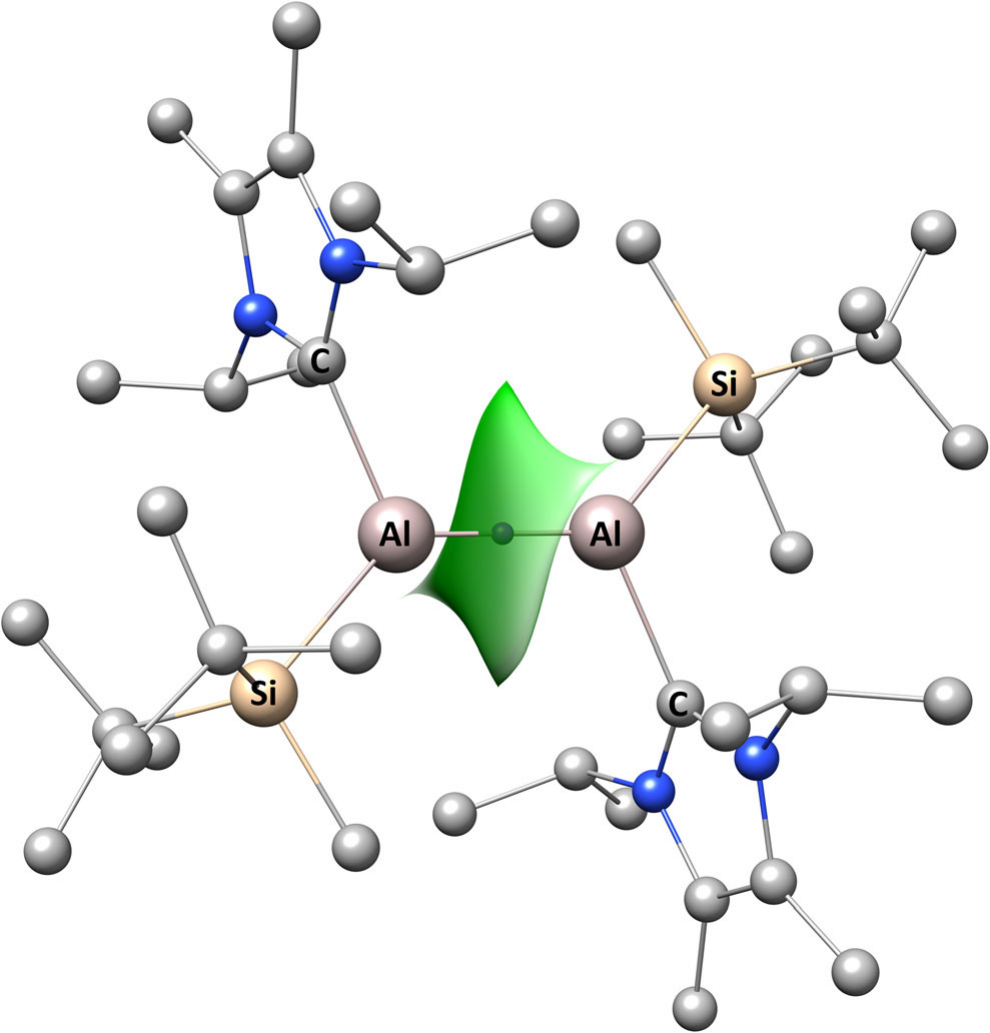
Basin of the non-nuclear attractor (NNA) of the Al=Al bond in dialumene. Calculations were carried out at the DFT(M06-L)/6-311++G(d,p) computational level

The BCP shows a high *ρ*_(3,−1)_(*r*) value for the B=B bond, 0.152 *e*/au^3^, and a low negative value for the Laplacian, −0.308 *e*/au^5^. This indicates that the B=B bond is covalent in character, in agreement with the NBO and ELF results. In the dialumene molecule, two nuclear attractors (Al atoms) are connected by two BCPs and one (3,−3) point that is not related to the nuclear position and is called a non-nuclear attractor (NNA). Similar NNAs were obtained for the M–M bond by Li et al. [39] for (η^5^-C_5_H_5_)_2_M_2_ (M = Be, Mg, Ca) molecules. The basin associated with NNA is shown in Fig. 4. The population of the pseudoatom associated with NNA is 0.937 *e*. The electron densities of the two BCPs are smaller than that for the NNA and range between 0.055 *e*/au^3^ (B3PW91) and 0.058 *e*/au^3^ (M06-2X). The negative value of ∇^2^*ρ*_(3,−1)_(*r*) is near to zero and ranges between −0.027 *e*/au^5^ (B3LYP) and −0.007 *e*/au^5^ (M06-L). For the Ga=Ga and In=In bonds, only a single (3,−1) critical point was localized, similar to the B–B bond. The values of *ρ*_(3,−1)_(*r*) for the Ga=Ga and In=In bonds (0.063 *e*/au^3^ and 0.046 *e*/au^3^, respectively) are similar to that for the Al=Al bond and are both much lower than the value obtained for the B=B bond. The values of ∇^2^*ρ*_(3,−1)_(*r*) are very small and positive, which suggests that the bonding character changes upon shifting from B to In atoms. However, it is worth remembering that those values were calculated using the ECPs for Ga and In. The sign of ∇^2^*ρ*_(3,−1)_(*r*) suggests that electron density is concentrated around the BCP of B=B and Al=Al and is depleted in the Ga=Ga and In=In bonds. Therefore, from an electron density perspective, the covalent character of the homoatomic T=T bond in the compound diminishes due to decreasing electron density and an increasing Laplacian as the atomic size of T increases.

The delocalization index, DI, is a quantitative measure of the number of electron pairs that are delocalized between two atomic basins. It is often considered to be equivalent to the topological bond order [40]. DI values for the molecules of interest are presented in Table 6. For the B=B, Ga=Ga, and In=In bonds, the index gradually decreases from 1.230 to 1.055 to 0.916, respectively. Those values suggest the presence of a single bond rather than a typical double bond. Similar calculations performed for the C_2_H_4_ molecule at the DFT(M06-L)/6-311++G(d,p) computational level yield a DI of 1.894. For the Al=Al bond with a NNA localized on the bond, we cannot interpret the DI as simply being equivalent to the topological bond order. All possible values for delocalization between the pseudoatom and the atomic basins are presented in Table 6. The number of electron pairs exchanged between the atomic basin of Al and the pseudoatom ranges between 0.451 (B3LYP) and 0.474 (PBE0) depending on the level of theory applied. Less electron density is involved in the delocalization between the two atomic basins of Al; the value of DI ranges between 0.467 (M06-L) and 0.588 (B3LYP).

**Table 6:** Delocalization indices (DI) for T=T bonds (T = B, Al, Ga, In) and T–NNA interactions

Bond/interaction	B	Al					Ga	In
	M06-L	B3LYP	B3PW91	PBE0	M06-L	M06-2X	M06-L	M06-L
T=T	1.23	0.588	0.553	0.519	0.467	0.531	1.055	0.916
T–NNA	–	0.451	0.462	0.474	0.473	0.452	–	–
NNA–T	–	0.451	0.462	0.474	0.473	0.452	–	–

Calculations were performed using wavefunctions approximated with DFT employing various electron density functionals and the 6-311++G(d,p) basis set. Calculations for the B, Ga, and In derivatives were performed at the DFT(M06-L)/6-311++G(d,p) computational level

NNA non-nuclear attractor

Since the results for the Ga and In derivatives were obtained with the effective core potential (ECP) approximation, the qualitative influence of the type of ECP applied on the NBO, *η*(*r*), and *ρ*(*r*) results should be considered. Hence, the calculations were repeated using three additional ECPs – ECP10MDF, ECP28MDF, and CRENBL – and selected results of the calculations are presented in Table 7. The populations of the atomic cores of Ga and In were found to be 9.50 *e*, 9.94 *e* (CRENBL) and 17.79 *e*, 17.88 *e* (ECP10MDF, ECP28MDF), respectively.

**Table 7:** Electron occupancies for the σ and π bonds, Wiberg bond indices (WBIs), basin populations, $\overline{N}$ (*e*) values for the T=T (T = Ga, In) bonding basins, their standard deviations (*σ*), and *ρ*_(3,−1)_(*r*) and ∇^2^*ρ*_(3,−1)_(*r*) values for (3,−1) critical points of the T=T bonds

Parameter	ECP			
	Ga (ECP10MDF)	Ga (CRENBL)	In (ECP28MDF)	In (CRENBL)
Natural bond orbital				
σ bond	1.85	1.85	1.76	1.76
π bond	1.70	1.69	1.69	1.68
WBI	1.52	1.54	1.35	1.37
Electron localization function				
*V*_1_(T,T)	1.87	2.23	1.99	1.93
*V*_2_(T,T)	1.87	2.24	1.98	1.92
* σ*	1.11 1.11	1.22 1.22	1.16 1.16	1.13 1.13
Electron density				
* ρ*_(3,−1)_(*r*) (*e*/au^3^)	0.065	0.058	0.047	0.045
∇^2^*ρ*_(3,−1)_(*r*) (*e*/au^5^)	−0.011	0.036	0.033	0.027

Calculations were performed with ECP10MDF or CRENBL for Ga-substituted dialumene and ECP28MDF or CRENBL for the In-substituted dialumene along with the 6-311++G(d,p) basis set

The NBO populations of the Ga=Ga and In=In σ bonds were found to be 1.85 *e* and 1.76 *e*, respectively, using the ECP10MDF/ECP28MDF and CRENBL approximations. A small decrease in electron occupancy is apparent when those values are compared with those calculated for the σ bonds using LANL2DZ (1.89 *e* and 1.82 *e*). Lower values were also obtained using the ECP10MDF and CRENBL approximations for the π bond of the Ga derivative: 1.69 *e* and 1.70 *e*, respectively. For the In derivative, the populations of the π bond were found to be 1.68 *e* and 1.69 *e*, respectively, using the ECP28MDF and CRENBL approximations. Analysis of the WBIs for the Ga and In derivatives revealed smaller values than those obtained using LANL2DZ. Thus, the influence of the ECP type on the NBO results is insignificant, and the results obtained with the LANL2DZ pseudopotential seem to be reliable.

**Fig. 5:**
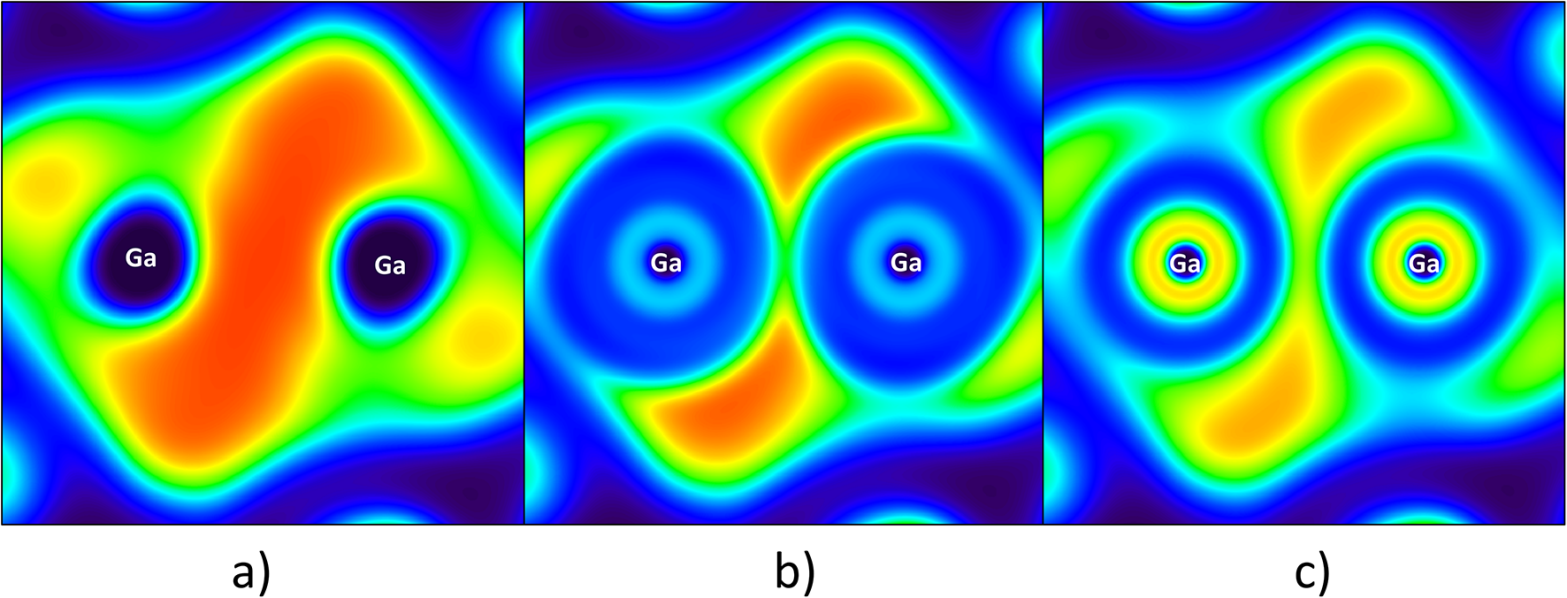
2D ELF plots for the Ga derivative, as calculated at the DFT(M06-L)/6-311++G(d,p) + ECP computational level, where the ECP is LANL2DZ (**a**), CRENBL (**b**), or ECP10MDF (**c**). The Ga=Ga bond is in the plane of the plot

The type of approximation used for the effective core potential has a significant influence on the results obtained for the topology of the ELF. The following discussion will concentrate on the T=T (T = Ga, In) molecular fragment. 2D ELF maps for the Ga atoms that were calculated using the CRENBL and ECP10MDF pseudopotentials are shown in Fig. 5b and c. The effect of the approximation adopted for the core regions can be observed as changes to the local ELF topology for the Ga=Ga bond. Instead of the single *V*(Ga,Ga) attractor obtained using the LANL2DZ ECP (see Fig. 5a), two *V*_*i*=1,2_(Ga,Ga) bonding attractors are found using CRENBL and ECP10MDF. The value of the ELF also depends on the ECP used, and it decreases for the *V*_*i*=1,2_(Ga,Ga) and *V*_*i*=1,2_(In,In) attractors in the order 0.939 (LANL2DZ) > 0.911 (CRENBL) > 0.841 (ECP10MDF) and 0.968 (LANL2DZ) > 0.878 (CRENBL) > 0.865 (ECP28MDF), respectively. Detailed analysis of the ELF distributions obtained with ECP10MDF, ECP28MDF, and CRENBL showed that the (3,+1) CP along the imaginary line joining the Ga nuclei has an ELF value of 0.528 (CRENBL) or 0.645 (ECP10MDF). Similar analysis performed for the In=In bond also showed that the (3,+1) CP had an ELF value of 0.445 (CRENBL) or 0.405 (ECP10MDF) – slightly smaller than the ELF values for the *V*_*i*=1,2_(In,In) attractors. Analysis of the ELF field using the LANL2DZ pseudopotential revealed that the (3,−1) CP situated along the imaginary line joining the nuclei had an ELF value of 0.528 (Ga) or 0.645 (In). In both cases, the values obtained reveal that calculations performed on cores with fewer electrons treated explicitly yield reduced electron localization in the T=T bonding region.

Total populations for the *V*_*i*=1,2_(Ga,Ga) basins (Table 7) are especially dependent on the pseudopotential applied; the values are 3.74 *e* using ECP10MDF and 4.47 *e* using CRENBL, which are higher than that obtained using LANL2DZ (3.58 *e*). Higher values were also obtained using ECP28MDF and CRENBL for the *V*_*i*=1,2_(In,In) basins: 3.97 *e* and 3.85 *e*, respectively. However, these results are rather similar. The standard deviations of the basin populations are close to those calculated using LANL2DZ (see Table 3). Thus, the effect of the ECP used on the ELF does not contradict the conclusions of our analysis performed using the LANL2DZ pseudopotential for the Ga and In atoms. Although the total basin populations for *V*_*i*=1,2_(Ga,Ga) and *V*_*i*=1,2_(In,In) differ from those calculated at the LANL2DZ level, the Ga=Ga and In=In bonds are still found to have double-bond character.

The effect of the choice of ECP on the topology of the *ρ*(*r*) field was also been studied in relation to the *ρ*_(3,−1)_(*r*) and ∇^2^*ρ*_(3,−1)_(*r*) values calculated for the Ga=Ga and In=In bonds. No significant influence of the particular ECP chosen was observed for the *ρ*_(3,−1)_(*r*) values of either bond, since similar values were obtained regardless of the ECP applied: 0.058–0.065 *e*/au^3^ (Ga=Ga) and 0.045–0.047 *e*/au^3^ (In=In). A more significant effect on the ∇^2^*ρ*_(3,−1)_(*r*) values was noted, as using ECP10MDF resulted in a value with a different sign (−0.011 *e*/au^5^) for the Ga-containing molecule. The Laplacian for the Ga=Ga BCP was smaller when calculated with the smallest ECP core (ECP10MDF) than with LANL2DZ. When more of the core electrons are replaced by pseudopotentials (i.e., when CRENBL is used), a higher ∇^2^*ρ*_(3,−1)_(*r*) value is obtained. Thus, the character of the Ga=Ga bond is uncertain at this level of calculation. Analysis of the data obtained for the In-containing molecule showed that a positive value of ∇^2^*ρ*_(3,−1)_(*r*) was obtained in all cases, so the ECP type did not have a significant impact on the *ρ*(*r*) topology. The value of ∇^2^*ρ*_(3,−1)_(*r*) given by ECP28MDF or CRENBL was smaller (by 0.007 or 0.013 *e*/au^5^, respectively) than that given by LANL2DZ.

## Conclusions

Application of the NBO method to and topological analyses of the *η*(*r*) and *ρ*(*r*) fields for the newly synthesized metalorganic dialumene and its derivatives enabled precise characterization of the local electronic structures of the T=T and T–C bonds (T = B, Al, Ga, In). We have shown that, in some respects, the molecular orbital theory approach and real functions yield complementary insights. Furthermore, the results highlight considerable differences between the local electronic structure of the nonmetal boron atoms and the local electronic structures of other metal atoms (Al, Ga, In) in these molecules.

NBO analysis revealed that the Al=Al, Ga=Ga, and In=In interactions each included both σ and π bonds, indicating that they all have double-bond character. However, the B–B interaction was characterized as including only σ bond. The WBIs for these bonds, which ranged from 1.55 (B=B) to 1.45 (In=In), did not point to any qualitative difference between B–B and other T=T (T = Al, Ga, In) bonds.

Analysis of the topology of the ELF showed that all of the T=T (T = B, Al, Ga, In) bonds were represented by bonding disynaptic attractors, *V*_*i*=1,2_(T,T). Thus, they have covalent character, with shared electron density. The B=B bond, with a population of 2.98 *e*, appears to be intermediate between a single and double bond, and its population is clearly smaller than those of the other T=T bonds: 3.44 *e* (Al), 3.58 *e* (Ga), and 3.86 *e* (In), which clearly have strong double-bond character. The largest population was obtained for the In=In bond. Thus, the results from the topological analysis of the ELF support the conclusions of the NBO analysis. Both methods yield results that highlight the differences in electronic structure between the bonds formed by the B, Al, Ga, and In atoms.

Topological analysis of the *ρ*(*r*) field showed a single bond critical point at the midpoints of the B–B, Ga=Ga, and In=In bonds and a non-nuclear attractor along the Al=Al bond. The population of the pseudoatom associated with the NNA was calculated as 0.937 *e*. The *ρ*_(3,−1)_(*r*) values for the Al=Al, Ga=Ga, and In=In bonds were found to be smaller than that for the B–B bond. The delocalization indices for the B=B, Ga=Ga, and In=In bonds gradually decreased from 1.230 to 1.055 to 0.916, respectively, suggesting the presence of a single bond rather than a typical double bond.
